# Exploring the role of sphingolipid machinery during the epithelial to mesenchymal transition program using an integrative approach

**DOI:** 10.18632/oncotarget.7947

**Published:** 2016-03-07

**Authors:** Anastasia Meshcheryakova, Martin Svoboda, Ammar Tahir, Harald C. Köfeler, Alexander Triebl, Felicitas Mungenast, Georg Heinze, Christopher Gerner, Philip Zimmermann, Markus Jaritz, Diana Mechtcheriakova

**Affiliations:** ^1^ Department of Pathophysiology and Allergy Research, Medical University of Vienna, Vienna, Austria; ^2^ Institute of Analytical Chemistry, University of Vienna, Vienna, Austria; ^3^ Mass Spectrometry Center, University of Vienna, Vienna, Austria; ^4^ Core Facility for Mass Spectrometry, Center for Medical Research, Medical University of Graz, Graz, Austria; ^5^ Section for Clinical Biometrics, Center for Medical Statistics, Informatics, and Intelligent Systems, Medical University Vienna, Vienna, Austria; ^6^ Nebion AG, Zürich, Switzerland; ^7^ Research Institute of Molecular Pathology, Vienna Biocenter, Vienna, Austria

**Keywords:** sphingolipid-related targets, sphingosine 1-phosphate/ceramide rheostat, multigene signature, epithelial to mesenchymal transition, lung cancer

## Abstract

The epithelial to mesenchymal transition (EMT) program is activated in epithelial cancer cells and facilitates their ability to metastasize based on enhanced migratory, proliferative, anti-apoptotic, and pluripotent capacities. Given the fundamental impact of sphingolipid machinery to each individual process, the sphingolipid-related mechanisms might be considered among the most prominent drivers/players of EMT; yet, there is still limited knowledge. Given the complexity of the interconnected sphingolipid system, which includes distinct sphingolipid mediators, their synthesizing enzymes, receptors and transporters, we herein apply an integrative approach for assessment of the sphingolipid-associated mechanisms underlying EMT program. We created the sphingolipid-/EMT-relevant 41-gene/23-gene signatures which were applied to denote transcriptional events in a lung cancer cell-based EMT model. Based on defined 35-gene sphingolipid/EMT-attributed signature of regulated genes, we show close associations between EMT markers, genes comprising the sphingolipid network at multiple levels and encoding sphingosine 1-phosphate (S1P)-/ceramide-metabolizing enzymes, S1P and lysophosphatidic acid (LPA) receptors and S1P transporters, pluripotency genes and inflammation-related molecules, and demonstrate the underlying biological pathways and regulators. Mass spectrometry-based sphingolipid analysis revealed an EMT-attributed shift towards increased S1P and LPA accompanied by reduced ceramide levels. Notably, using transcriptomics data across various cell-based perturbations and neoplastic tissues (24193 arrays), we identified the sphingolipid/EMT signature primarily in lung adenocarcinoma tissues; besides, bladder, colorectal and prostate cancers were among the top-ranked. The findings also highlight novel regulatory associations between influenza virus and the sphingolipid/EMT-associated mechanisms. In sum, data propose the multidimensional contribution of sphingolipid machinery to pathological EMT and may yield new biomarkers and therapeutic targets.

## INTRODUCTION

The perception that lipids play critical roles in many aspects of cell regulation uncovers fundamentally novel mechanisms and pathways to interfere with upon dysregulation in distinct diseased conditions. Among those are sphingolipids. Sphingolipids comprise a highly diverse and complex class of lipid molecules that, on the one side, have structural roles in the regulation of the fluidity and the sub-domain structure of the cellular lipid bilayers, while, on the other side, are active participants in intracellular and extracellular signaling, and thereby, play important roles in the regulation of a variety of vital cellular and biological processes. Among the most studied are cell migration, cell proliferation, cell death/apoptosis and morphogenesis. Aberrations/dysregulations of the mechanisms underlying these processes are known to be linked to several pathologies thus nominating sphingolipids as key players in diverse pathological processes including malignant transformation [1–7]; accordingly, various routes of the sphingolipid machinery might be therapeutically targeted.

**Figure 1:**
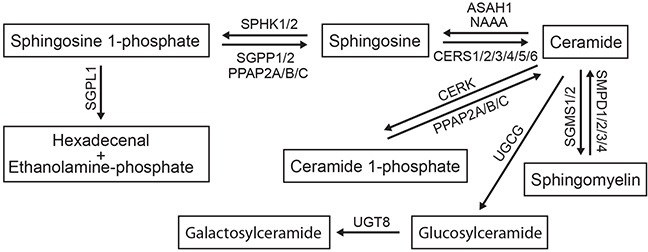
The sphingomyelin/salvage pathway The interconnected network of sphingolipid mediators composing the sphingomyelin/salvage pathway (also known as the sphingomyelin cycle) and the corresponding sphingolipid-modifying enzymes are shown. Detailed description of genes encoding the enzymes is provided in Results. An arrow indicates the direction of lipid conversion.

Sphingolipids are defined by the presence of a backbone sphingoid base that can be additionally modified — phosphorylated, acylated, glycosylated, or bridged to various headgroups through phosphodiester linkages [8] — by specific enzymes (Figure 1). The diverse lipid effector molecules, including ceramide (Cer) and its complex derivatives or its phosphorylated form C1P, sphingosine (Sph) and its phosphorylated derivative S1P, may act in various modes being actively incorporated in different cellular pathways. Upon formation within a cell, they may bind to intracellular target molecules and modulate their activity [9, 10]. Alternatively, the lipid mediators may be secreted from cells and then interact with specific cell surface receptors, as best documented for S1P through binding to specific G-protein coupled receptors S1PR1-5 (for recent reviews see [11, 12]). Among the plethora of metabolites, S1P and Cer have emerged as potent mediators generally functioning with opposing biological effects, and thus the S1P/Cer rheostat is considered as one of the decisive cellular factors to drive either proliferation or apoptosis (reviewed in [4]).

There are lines of evidence suggesting a fundamental and perhaps multistep involvement of bioactive sphingolipids and their synthesizing enzymes in almost every aspect of neoplastic cell behavior and the malfunctions of malignant disorders based on their capabilities to modulate/regulate such critical processes as cell proliferation, migration, angiogenesis, apoptosis, and acquisition of a multidrug resistance, immune response, and inflammation [13–17]. Recent evidences furthermore suggest that the sphingolipid machinery, in particular the SPHK1/S1P/S1PR axis, regulates the communication between cancer cells and the host organism and by this way promotes metastasis [18, 19]. In this regards, there is an additional body of evidence demonstrating that S1P is involved in many different types of fibrosis including the tumor-associated fibrosis by promoting either differentiation of stroma fibroblasts into myofibroblasts and/or their migration [18, 20] with TGFbeta being a key factor. Notably, the expression of S1P producing SPHK1 and of some S1PR family members can be triggered by TGFbeta [21, 22]. Given that, surprisingly limited information is available linking the sphingolipid mediators and the epithelial to mesenchymal transition (EMT) — a key developmental program that is considered as a critical cancer promoting process. Indeed, the activation of EMT program has been implicated as an important step in metastasis of the lung and other epithelial-derived cancers. Divergent factors have been described to be able to initiate EMT including among others combinations of growth factors and/or proinflammatory cytokines and/or chemokines (reviewed in [23, 24]). The most potent EMT triggering cocktails have, however, clear cell type-specific characteristics; this emphasizes the variety of potential mechanisms controlling the transformation programs.

Primary lung cancer is among the most common malignancies in males and females worldwide; an over one in four cancer death is due to lung cancer [25, 26]. Non-small cell-lung cancer (NSCLC) accounts for majority of cases (85%) and further sub-divided to adenocarcinoma (40%), squamous cell carcinoma (25-30%) and large cell carcinomas (10-15%) [27]. NSCLC is characterized by high metastatic potential; metastases named synchronous can be diagnosed even before the primary tumor, while the metachronous metastases appear after treatment of the primary tumor [26]. Notably, *in vitro* A549 cell-based EMT model with TGFbeta being the most prominent and studied EMT trigger [28] can be used to investigate the underlying mechanisms of cellular transformation and metastasis in NSCLC.

Herein we tested the hypothesis that the sphingolipid-associated events are among the mechanisms underlying the EMT program in lung cancer. Complexity of the sphingolipid network and signaling resulting in multifaceted contribution of the sphingolipid machinery to diverse pathways and mechanisms dictates the necessity of the implementation of more integrative, systems biology-based approaches for analysis and overview picture. In this study we applied a multigene signature-based profiling approach assessing the sphingolipid/EMT-associated gene network combined with analysis of sphingolipid mediators, at first, in the EMT cell-based model followed by gene network analysis and reconstruction of associated biological pathways and regulators. Next, on the basis of defined sphingolipid/EMT-associated signature-based profile we performed alignment with publicly available transcriptomics data sets and assessed under which perturbations and diseased conditions the sphingolipid/EMT-associated signature might occur. Such comprehensive analysis thus allowed us to propagate the *in vitro* cell-based findings and conclusions to novel aspects of disease pathobiology.

## RESULTS

### Differential EMT-associated phenotypic alterations triggered by TGFbeta, TNFalpha and their combination in A549 cells

**Figure 2:**
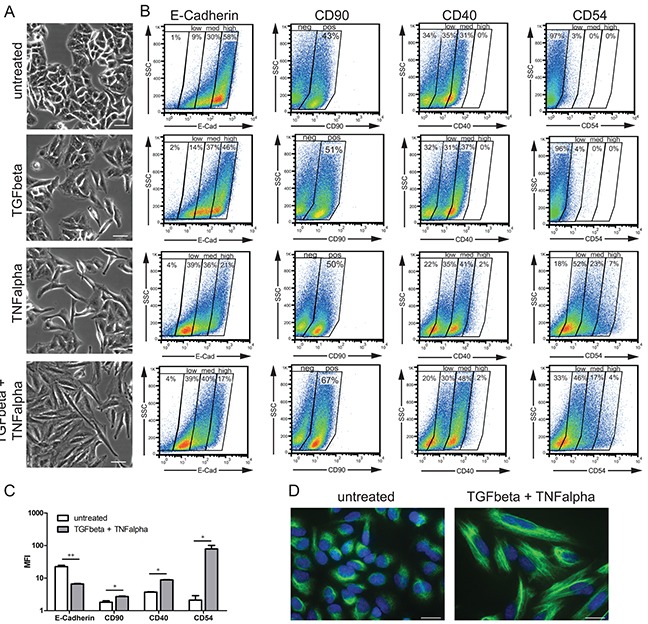
Monitoring of EMT-associated phenotypic alterations **A.** Phase-contrast images of A549 cells at epithelial stage (untreated) and mesenchymal/fibroblast-like stage (TGFbeta, TNFalpha, and TGFbeta + TNFalpha) are shown. Scale bar: 50 μm. Pictures are representative of three independent experiments. **B.** Cell surface expression of EMT markers (E-Cadherin and CD90) and inflammation-associated molecules (CD40 and CD54/ICAM1) at epithelial and mesenchymal/fibroblast-like stages assessed by flow cytometry is shown next to the corresponding microscopic images. Based on the staining intensities, gates were set to discriminate between negative, low, medium, and high expression levels (E-Cadherin, CD40 and CD54) or between negative and positive (CD90). Results are representative of two independent experiments. **C.** Bar graph indicates mean fluorescence intensity (MFI) values for the indicated EMT markers at epithelial stage (untreated) and mesenchymal/fibroblast-like stage (TGFbeta + TNFalpha). Values are displayed as means ± SD of two independent experiments. Significance was assessed by two-tailed *t* test; * p<0.05 and ** p<0.01. **D.** Images of immunofluorescent staining of vimentin (green) in untreated cells and cells treated with TGFbeta + TNFalpha; cell nuclei were stained with DAPI (blue). Scale bar: 20 μm. Pictures are representative of two independent experiments.

To study the EMT process in a cell-based model, A549 cells — human alveolar epithelial cells from adenocarcinoma — were stimulated with TGFbeta (2 ng/ml), TNFalpha (12.5 ng/ml), their combination or left untreated; the characterization of EMT was performed by microscopy, flow cytometric analysis, immunofluorescent assay, and gene expression profiling (see Material and Methods). To monitor the EMT process we first performed microscopic evaluation of cell morphology at 48 h time point upon stimulation (Figure 2A). In comparison to untreated cells, which showed classical cobblestone epithelial cell morphology, all three stimulation conditions, as anticipated, resulted in acquisition of spindle-shaped, fibroblast-like mesenchymal phenotype; the strongest effect was thereby observed for TGFbeta + TNFalpha. Furthermore, the flow cytometry-based monitoring (Figure 2B and 2C) revealed strongest downregulation of the epithelial cell adhesion marker E-Cadherin (also known as CDH1) following TGFbeta + TNFalpha treatment, whereby a predominantly E-Cadherin^high^ population was converted into a predominantly E-Cadherin^low/medium^ population (Figure 2B). The loss of surface E-Cadherin expression was accompanied by upregulation of the fibroblast marker CD90 (also known as THY1) upon stimulation with TGFbeta + TNFalpha. Thus, for both molecules the strongest shift to EMT was determined for the combination of cytokines. Given the inclusion of the pro-inflammatory stimulus TNFalpha in this experiment, we further assessed the expression levels of TNFalpha-dependent, inflammation-associated molecules CD40 (also known as TNFRSF5) and CD54 (also known as ICAM1). CD40 was detected on unstimulated cells at epithelial stage and showed moderate upregulation of expression at the mesenchymal/fibroblast-like stage upon stimulation with TNFalpha or TGFbeta + TNFalpha. In contrast, CD54 was neither expressed on untreated epithelial nor TGFbeta-treated A549 cells, whereas showed strong induction upon treatment with TNFalpha or TGFbeta + TNFalpha. Next, we used immunofluorescence assay to determine cellular distribution of vimentin, an additional canonical EMT marker, in cells before and after treatment with TGFbeta + TNFalpha. Upon treatment, vimentin was redistributed from perinuclear zone to form intermediate filaments of cytoskeleton, thus accentuating elongated, mesenchymal/ fibroblast-like shape of the cells (Figure 2D).

Taken together, the results demonstrate that the combination of both cytokines provides the strongest stimulus to drive the EMT process in A549 cells, thus, the TNFalpha treatment potentiates the TGFbeta-triggered EMT in A549 cell-based model.

### A multigene signature approach to assess transcriptional profiles in the context of particular biological process

We apply a multigene signature approach as a starting point to characterize on the gene expression level (i) the EMT program and (ii) the interconnected process of sphingolipid turnover and signaling merged with LPA signaling. Selection of genes composing the multigene signature is knowledge- and biology-driven, whereby such expert-designed gene signature covers the most relevant genes of particular biological pathway or process; certainly, this selection is influenced by previous published work and process relevance, and in this sense is biased towards previous knowledge. This approach represents relatively new way of addressing transcriptional profiles, gene networks and methodologically has incontestable advantage of the real-time PCR-based analysis that ultimately provides quantitative and reproducible results which do not need further methodological validation. The compositions of the herein created and applied signatures such as the EMT-associated 23-gene signature and the sphingolipid-associated 41-gene signature are specified in Material and Methods.

### EMT-associated signature allows monitoring of the EMT process on the gene expression level

**Figure 3:**
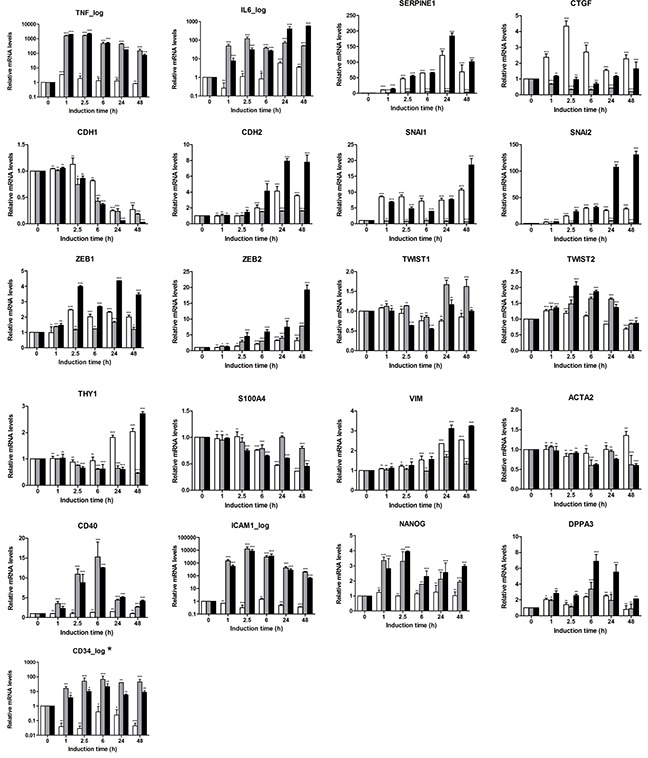
Characterization of EMT program triggered in A549 cells by gene expression profiling using the EMT-related 23-gene signature Expression pattern of EMT-related genes was assessed in kinetics up to 48 h using real-time PCR-based expression profiling. Relative expression was calculated using the ΔΔC_T_ method. Levels of mRNA were normalized to HKG and shown relative to unstimulated cells (0 h time point). Data are displayed as means ± SD of two independent runs. Significance was assessed by two-way analysis of variance (ANOVA); * p<0.05, ** p<0.01, *** p<0.001, ns, not significant. Representative results of three independent experiments are shown (Supplementary Table S1). Color code of bar chart: *white*, stimulated with TGFbeta; *grey*, stimulated with TNFalpha; *black*, stimulated with TGFbeta + TNFalpha. Low expressing genes (Ct>30) are indicated by asterisk at the gene name. Genes with Ct>35 were classified herein as *not expressed* and are not shown (*SNAI3* and *CD133/PROM1*).

The EMT-associated multigene signature was applied to characterize the early EMT-associated regulators (1 h, 2.5 h and 6 h time points) and the transcriptional outcome of the transdifferentiation process (24 h and 48 h time points) in the A549 cell-based EMT model (Figure 3). Both TNFalpha and TGFbeta triggered strong induction of the corresponding responsive genes. Thus, *TNF* itself showed a quick response with about 1600-fold upregulation determined at 1 h; upon transdifferentiation, the mesenchymal/fibroblast-like cells still showed upregulated mRNA levels. *IL6* showed strong response at 1 h of induction and was further upregulated with the progression of the EMT process upon TNFalpha + TGFbeta treatment. For the control genes *SERPINE1* and *CTGF*, the expression profiling confirmed the TGFbeta-dependent upregulation. Expression profiling further revealed that *CDH1* (also known as *E-Cadherin*) mRNA levels were strongly downregulated within the EMT process triggered by all three stimulation conditions with the strongest effect observed for the combination of cytokines (0.03-fold at 48 h). An opposing behavior was detected for *CDH2* which expression was further upregulated upon stimulation. Within the *SNAI* gene family, *SNAI1* and *SNAI2* were strongly upregulated at the 24 h and 48 h time points (*SNAI1* < *SNAI2*), showing a TGFbeta-dependent transcriptional regulation which was further potentiated by TNFalpha; the synergistic effect of TNFalpha on TGFbeta-triggered *SNAI2* is rather indirect. *SNAI3* was not expressed in the A549 cell-based model. Among the *ZEB* family members, the moderate upregulation of *ZEB1* (3.5-fold at 48h by TGFbeta + TNFalpha) and the strong upregulation of *ZEB2* (19.2-fold at 48h by TGFbeta + TNFalpha) were detected; for both genes the TGFbeta + TNFalpha treatment resulted in an additive effect. *TWIST1/2* mRNAs showed only minor regulation. The fibroblast marker *THY1* (CD90), which was as well analyzed on the protein level by flow cytometry (Figure 2B), showed moderate mRNA upregulation. When analyzing cytoskeleton components we found minor/moderate upregulation of *VIM* mRNA levels and no/minor regulation of *ACTA2* (encoding alpha-SMA) on the transcriptional level. Assessment of the inflammation-associated, TNFalpha-dependent genes revealed strong upregulation of *CD40* mRNA at the early time points with the maximal expression levels detected at 6 h upon stimulation with TNFalpha or combination, and moderate upregulation determined at the mesenchymal/fibroblast-like stage (24 h and 48 h). Regarding the *ICAM1* mRNA expression levels, we observed a strong induction by TNFalpha or combination at 2.5 h of stimulation; strongly enhanced *ICAM1* mRNA levels were also maintained at 24 h and 48 h (63.6-fold at 48 h for TGFbeta + TNFalpha). For the plasticity/pluripotency-associated genes we observed minor to moderate upregulation of *NANOG* and *DPPA3* upon triggering of the EMT program by TNFalpha and/or TNFalpha + TGFbeta; *CD34*, categorized as low expressing gene, was similarly upregulated; *PROM1* was not expressed.

### EMT-associated alterations in sphingolipid machinery assessed by expression profiling

**Figure 4:**
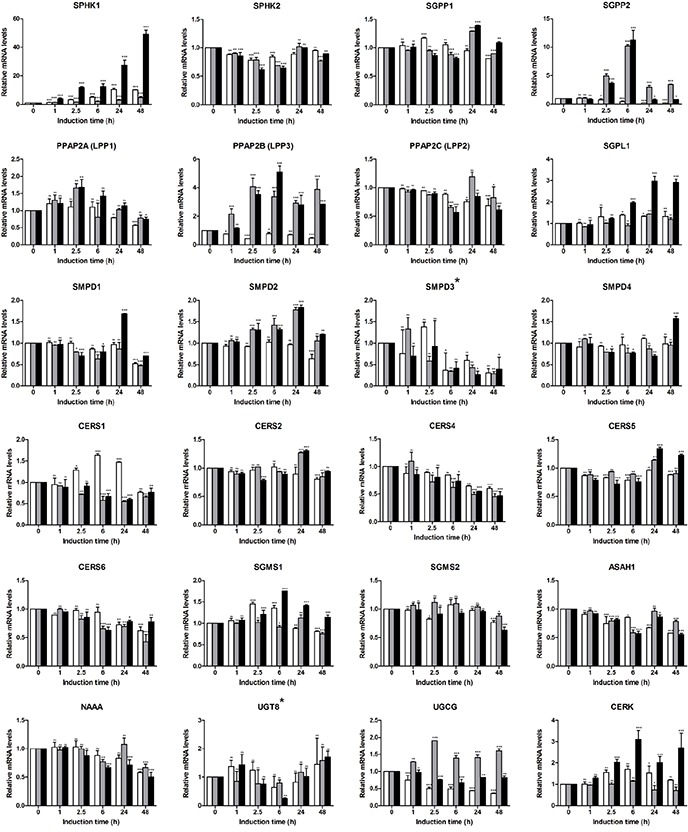
Expression profiling of sphingolipid-related genes in A549-based EMT model using sphingolipid-associated 41-gene signature Expression levels of genes comprising the multigene signature were assessed by real-time PCR-based profiling and represented as relative expression values using the ΔΔCT method. Levels of mRNA were normalized to HKG and shown relative to unstimulated cells (0 h time point). Data are displayed as means ± SD of two independent runs. Significance was assessed by two-way analysis of variance (ANOVA); * p<0.05, ** p<0.01, *** p<0.001, ns, not significant. Representative results of three independent experiments are shown (Supplementary Table S1). Color code of bar chart: *white*, stimulated with TGFbeta; *grey*, stimulated with TNFalpha; *black*, stimulated with TGFbeta + TNFalpha. Low expressing genes (Ct>30) are indicated by asterisk at the gene name. Genes with Ct>35 were classified as not expressed and are not shown (*ENPP2*, *CERS3*).

We next used the sphingolipid-related 41-gene signature to assess the EMT-associated gene alterations and define the gene patterns characteristic for epithelial and mesenchymal/fibroblast-like cell stages (Figure 4). With respect to S1P-producing enzymes, we observed strong upregulation of *SPHK1* with an expression pattern showing a synergistic effect of the two stimuli (49-fold at 48 h for TGFbeta + TNFalpha); *SPHK2* showed a stable expression pattern. For S1P-metabolizing genes, among the phosphatases *SGPP2* showed a clear TNFalpha-dependent upregulation at the early time points (10.2-fold at 6 h) which was maintained at lower levels at the late time points (3.5-fold at 48 h); addition of TGFbeta reduced the TNFalpha-mediated effect at the mesenchymal/fibroblast-like stage. *PPAP2B* mRNA showed continuous TNFalpha- and TGFbeta + TNFalpha-triggered upregulation. *SGPL1* mRNA showed only minor/moderate upregulation by TGFbeta + TNFalpha at mesenchymal/fibroblast-like cell stage. Within the *SMPD* family, genes showed either no or minor transcriptional regulation. Similarly, among CERSs, no or minor regulation was detected, with *CERS4* and *CERS6* being downregulated. For Cer-utilizing enzymes, the expression of genes encoding *SGMS1/2*, *ASAH1* and *UGT8* appeared not to be regulated, while *NAAA* mRNA levels showed minor/moderate downregulation. *UGCG* mRNA expression was reduced by TGFbeta; this TGFbeta effect was compensated by TNFalpha. The C1P-producing CERK showed moderate upregulation upon TGFbeta + TNFalpha treatment. Within the S1PR family, we found moderate upregulation of *S1PR1*, *S1PR2*, *S1PR3* and *S1PR5* at 48 h time point. For all indicated genes, the combination of TGFbeta and TNFalpha showed strongest effects. *S1PR4* showed minor downregulation in response to TGFbeta + TNFalpha. Differential expression patterns were also observed within the family of LPARs, with *LPAR2*, *LPAR4* and *LPAR5* being upregulated, while *LPAR1* and *LPAR3* being downregulated; *LPAR6* showed no/minor expression change. In respect of transporters, we observed an upregulation of *ABCA1* mRNA levels with an additive effect of TGFbeta and TNFalpha (5.3-fold at 48 h with TGFbeta + TNFalpha); in contrast, expression levels of *ABCG2* were strongly downregulated (0.1-fold at 48 h with TGFbeta + TNFalpha). *SPNS2* showed a pattern of an early responsive gene with maximal expression levels detected at 2.5 h upon stimulation with TGFbeta + TNFalpha, while at 48 h time point the *SPNS2* mRNA levels were downregulated even below the epithelial stage/basal level. An overview of the EMT-driven differentially expressed genes (fold change ≥2 for upregulated and ≤0.5 for downregulated genes at 48 h), that are common or unique across three treatment conditions, is given by a Venn diagram (Supplementary Figure S1).

As outcome of the signatures-based profiling we define the sphingolipid/EMT-associated 35-gene signature composed of transcriptionally regulated genes (fold change ≥2 for upregulated and ≤0.5 for downregulated genes at 48 h; TGFbeta + TNFalpha stimulation condition) which is characteristic for the EMT-driven transcriptional switch.

### Uncovering gene-gene associations characterizing the interplay between the sphingolipid machinery and EMT-associated markers during and at the end point of EMT transition

**Figure 5:**
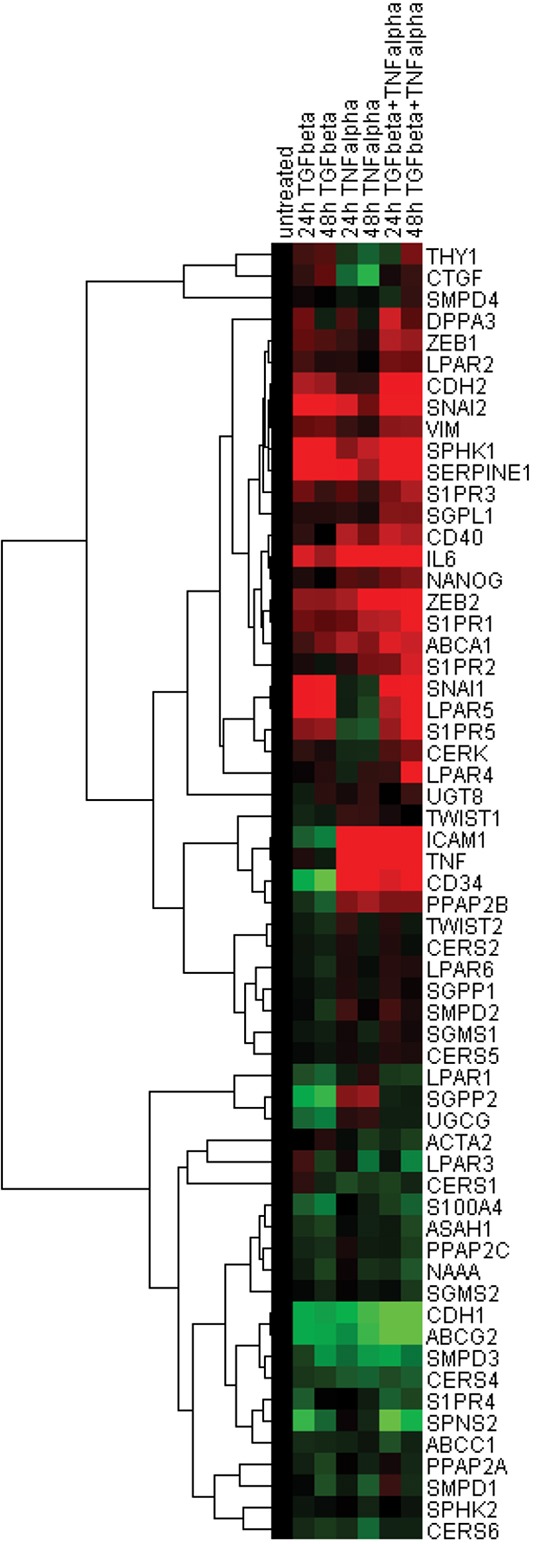
Hierarchical clustering of expression data sets Pearson uncentered hierarchical clustering was applied on log2 transformed fold-change values derived from EMT- and sphingolipid-associated multigene signatures (24 h and 48 h time points; Cluster/TreeView programs). Color code: *red*, upregulation and *green*, downregulation according to Cluster/TreeView [89].

For further visualisation and interpretation of these expression profiles, we applied a hierarchical cluster analysis on the basis of expression values of genes comprising the EMT- and sphingolipid-associated signatures across three stimulation conditions at the mesenchymal/fibroblast-like stage (24 h and 48 h time points, Figure 5). The resulting two main clusters are clearly associated with a distinct gene expression profile: cluster I was built by genes upregulated upon EMT as well as by genes showing no or minor regulation, and cluster II was comprised of genes downregulated at the mesenchymal/fibroblast-like stage. Among the upregulated genes within cluster I, the S1P and LPA receptor family members (*S1PR1*/*2*/*5* and *LPAR2*/*4*/*5*), genes encoding the S1P-producing *SPHK1* and C1P-producing *CERK*, the S1P-degrading *SGPL1* as well as the S1P/LPA/C1P phosphatase *PPAP2B*, and the transporter *ABCA1* were clustered together with the classical EMT markers such as *CDH2*, *SNAI1/2*, *ZEB1/2*, and *VIM*, pluripotency genes *NANOG*, *DPPA3*, and *CD34* as well as inflammation-associated molecules *TNF*, *IL6*, *ICAM1*, and *CD40*. Cluster II was build up around the S1P and LPA receptor family members (*S1PR4* and *LPAR1/3*), genes encoding the S1P/LPA/C1P phosphatases *SGPP2* and *PPAP2A/2C*, genes encoding the Cer-producing *CERS1/4/6* and *SMPD1/3*, the Cer-utilizing *UGCG*, *ASAH1*, *NAAA*, and *SGSM2*, transporters *ABCC1*, *ABCG2* and *SPNS2* in close association with classical EMT marker *CDH1*.

**Figure 6:**
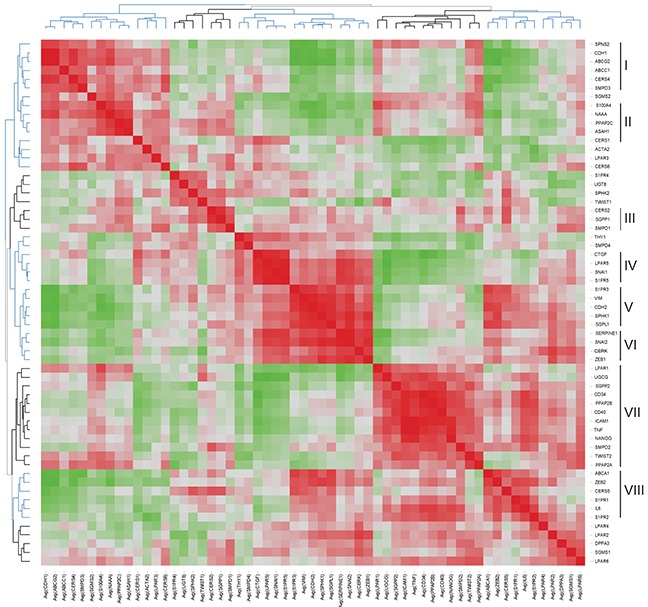
Heat map representing the correlation matrix for EMT- and sphingolipid-associated genes Pearson correlation matrix was calculated across all time points and all stimulation conditions (Supplementary Table S2). The corresponding correlation coefficients were subjected to unsupervised hierarchical clustering using Euclidean distance measurement (average linkage clustering). Each colored square illustrates the gene-gene interaction; color code: red, positive correlation; green, negative correlation; the color intensity represents the correlation strength. Main clusters of co-modulated markers are indicated (right).

Hierarchical clustering of the gene-gene correlation matrix revealed eight main gene clusters highlighting close associations between EMT markers and sphingolipid-related genes; the corresponding heat map of associated correlation coefficients is illustrated in Figure 6 and the underlying correlation matrix is given in Supplementary Table S2. Cluster I (*SPNS2-CDH1-ABCG2-ABCC1-CERS4-SMPD3*) included genes downregulated by EMT process and showed the closest association between CDH1 and ABCG2 (correlation coefficient 0.985; FDR-adjusted p<0.001). Genes within Cluster II (*S100A4-NAAA-PPAP2C-ASAH1-CERS1*) and Cluster III (*CERS2-SGPP1-SMPD1*) showed no or minor regulations. Genes that showed the TGFbeta-dependent upregulation were grouped in Cluster IV (*CTGF-LPAR5-SNAI1-S1PR5*) with the highest degrees of association between *LPAR5* and *SNAI1* (correlation coefficient 0.918; FDR-adjusted p<0.001), between *S1PR5* and *SNAI1* (correlation coefficient 0.947; FDR-adjusted p<0.001) and between *LPAR5* and *S1PR5* (correlation coefficient 0.941; FDR-adjusted p<0.001). Cluster V (*S1PR3-VIM-CDH2-SPHK1-SGPL1*) and Cluster VI (*SERPINE1-SNAI2-CERK-ZEB1*) comprised the subset of upregulated genes and showed the closest association between *VIM* and *CDH2* (correlation coefficient 0.925; FDR-adjusted p<0.001) and *SERPINE1* and *SNAI2* (correlation coefficient 0.953; FDR-adjusted p<0.001). The large Cluster VII (*LPAR1-UGCG-SGPP2-CD34-PPAP2B- CD40-ICAM1-TNF-NANOG-SMPD2-TWIST2-PPAP2A*) was grouped around upregulated genes encoding the TNF-responsive inflammation-associated molecules, pluripotency markers and genes of sphingolipid machinery with the highest degrees of association between *ICAM1* and *TNF* (correlation coefficient 0.967; FDR-adjusted p<0.001) or *ICAM1* and *CD40* (correlation coefficient 0.909; FDR-adjusted p<0.001), or *ICAM1* and *PPAP2B* (correlation coefficient 0.904; FDR-adjusted p<0.001), *ICAM1* and *CD34* (correlation coefficient 0.914; FDR-adjusted p<0.001), *TNF* and *NANOG* (correlation coefficient 0.913; FDR-adjusted p<0.001), and *CD34* and *PPAP2B* (correlation coefficient 0.939; FDR-adjusted p<0.001). Within Cluster VIII (*ABCA1-ZEB2-CERS5-S1PR1-IL6-S1PR2*) the closest association was established between *S1PR1* and *IL6* (correlation coefficient 0.859; FDR-adjusted p=0.001).

### EMT-associated alteration of S1P/ceramide rheostat and of LPA species assessed by mass spectrometry-based analyses

**Figure 7:**
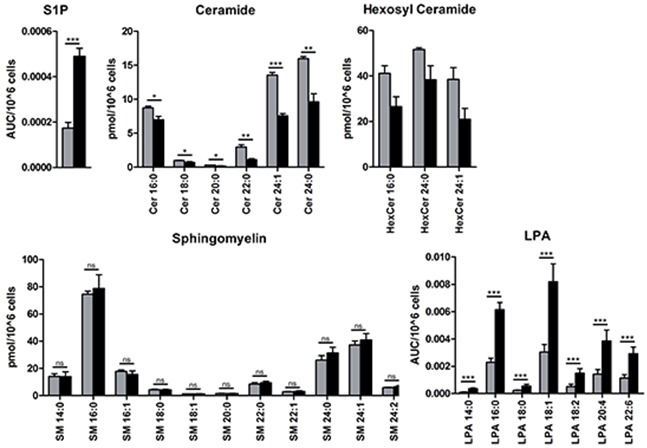
Mass spectrometry-based analysis of sphingolipid and LPA species Endogenous intracellular levels of S1P, ceramide (Cer), hexosyl ceramide (HexCer) and sphingomyelin (SM) as well as LPA were assessed as described in Material and Methods and displayed as area under the curve, AUC, per 10^6^ cells (S1P and LPA) or pmol per 10^6^ cells (Cer, HexCer and SM); species are indicated by the corresponding chain length. Color code of the bar chart: *grey*, epithelial cell stage; *black*, mesenchymal/fibroblast-like cell stage. Values are displayed as means ± SD of three independent experiments. Significance was assessed by two-tailed *t* test; * p<0.05, ** p<0.01, *** p<0.001, n.s., not significant.

Next, we assessed the levels of S1P, Cer, HexCer, and SM in A549 cells at epithelial stage and at the mesenchymal/fibroblast-like stage (48 h with TGFbeta + TNFalpha). We found an increase of intracellular S1P by about 2-fold at the mesenchymal/fibroblast-like stage compared to unstimulated cells concomitant with reduced Cer and HexCer levels; SM levels were not modulated (Figure 7). Given the overlap between turnover and signaling for sphingolipids and LPA, we furthermore assessed the levels of LPA species. A549 cells at the mesenchymal/fibroblast-like stage were characterized by increased levels of intracellular LPA when compared to those at the epithelial stage (Figure 7).

### Data-driven signature-associated linkage to canonical pathways and upstream regulators: gene network reconstruction

**Figure 8:**
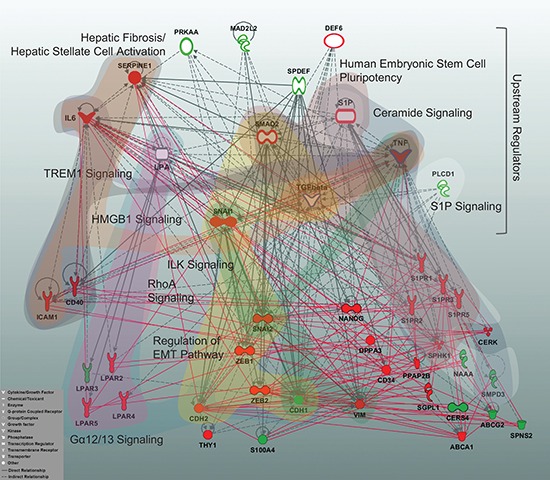
Multigene signature-based sphingolipid/EMT-associated gene network displaying the top Canonical Pathways and Upstream Regulators A reconstructed gene network was created using the Ingenuity Pathway Analysis Software (IPA) on the basis of EMT-driven differentially expressed genes (fold change ≥2 for upregulated and ≤0.5 for downregulated genes at 48 h TGFbeta + TNFalpha, Figures 3 and 4) and the top molecules from Upstream Regulators. Grey solid lines display the IPA-identified direct associations between molecules; grey dashed lines display the IPA-identified indirect associations between molecules. Additionally, statistically significant, herein defined biological associations assessed by correlation analysis (correlation coefficient >0.7; Supplementary Table S2) are displayed by red solid lines. Color code: *red fill*, upregulated gene from multigene signature; *green fill*, downregulated gene from multigene signature; *white fill*, IPA-predicted Upstream Regulator; *white fill with a red border*, IPA-predicted Upstream Regulator acting as activator; *white fill with a green border*, IPA-predicted Upstream Regulator acting as inhibitor; blue border highlights the Upstream Regulators used herein as stimuli. The top 10 IPA-based Canonical Pathways are highlighted by colored areas covering the corresponding pathway-attributed molecules; the IPA nomenclature was used to designate Canonical Pathways. Insert: the IPA-based description of symbols and relationships.

To summarize and expand the signature-driven information through a higher-level overview, we utilized an Ingenuity Pathway Analysis (IPA)-based ‘core analysis’ to align the sphingolipid/EMT-based data sets with IPA's Canonical Pathways and Upstream Regulators (complete lists of significant outcomes are given in Supplementary Table S3 and Supplementary Table S4). Furthermore, we reconstructed the interaction network on the basis of the sphingolipid/EMT genes showing the EMT-dependent regulation and IPA-derived Upstream Regulators (specified in Material and Methods) and visualized the 10-top Canonical Pathways by highlighting the corresponding pathway-attributed molecules (Figure 8). These Canonical Pathways are assigned to Ceramide Signaling, Sphingosine 1-phosphate Signaling, Gα12/13 Signaling, Human Embryonic Stem Cell Pluripotency, Regulation of the Epithelial-Mesenchymal Transition Pathway, ILK Signaling, TREM1 Signaling, Hepatic Fibrosis/ Hepatic Stellate Cell Activation, and HMGB1 Signaling and show overlaps. The applied strategy allowed us to dissect potential mechanisms, pathways and factors linking the sphingolipid machinery, the EMT program, pluripotency, and inflammation.

### Integrated analysis of the sphingolipid/EMT-associated gene alterations under diverse cell perturbations and diseased conditions using curated microarray data sets from public databases

We wanted to assess whether the herein identified sphingolipid/EMT-associated 35-gene signature, attributed to TGFbeta + TNFalpha stimulation condition, can be similarly detected in other cell-based models or treatment conditions and, importantly, in neoplastic tissues from patients with various types of lung malignancies and/or with other cancer types. We therefore examined the similarities with expression profiles across previously published microarray data sets using the GENEVESTIGATOR platform (Tables 1–4). The comparison is based on the measurement of a distance that quantified the degree of similarity between the defined signature profile and the transcriptional profile for a condition; the result of the comparison is a relative similarity index (see Methods).

**Table 1:** The 10-top-sphingolipid/EMT signature-linked cell-based models/conditions identified by GENEVESTIGATOR

Cell Lines_Pathological Cell Lines_Neoplastic Cell Lines_A549				
Position	Study	Rel. Sim.	Study Number	Reference
1	Influenza virus study 3 (A/pH1N1) / mock infected A549 cell sample	2.592	GSE31524	[90]
2	TGF-β study 5 (late) / untreated A549 cell sample	2.385	GSE17708	[91]
3	Influenza virus study 2 (A/H5N2) / mock infected A549 cell sample	2.076	GSE31524	[90]
4	TGF-β study 5 (intermediate) / untreated A549 cell sample	1.87	GSE17708	[91]
5	Influenza virus study 3 (A/H5N2) / mock infected A549 cell sample	1.786	GSE31524	[90]
6	Influenza virus (A/H5N2) / mock infected A549 cell sample	1.692	GSE31524	[90]
7	Influenza virus study 2 (A/pH1N1) / mock infected A549 cell sample	1.634	GSE31524	[90]
8	Influenza virus (A/pH1N1) / mock infected A549 cell sample	1.631	GSE31524	[90]
9	Influenza virus study 3 (A/H9N2) / mock infected A549 cell sample	1.562	GSE31524	[90]
10	Influenza virus study 2 (A/H1N1) / mock infected A549 cell sample	1.386	GSE31524	[90]
**Cell Lines_Pathological Cell Lines_Neoplastic Cell Lines_All**				
**Position**	**Study**	**Rel. Sim.**	**Study Number**	**Reference**
1	Influenza virus study 3 (A/pH1N1) / mock infected A549 cell sample	2.777	GSE31524	[90]
2	TGF-β study 5 (late) / untreated A549 cell sample	2.555	GSE17708	[91]
3	S. pneumoniae / mock infected Detroit 562 cell sample	2.443	GSE8527	[92]
4	Influenza virus study 2 (A/H5N2) / mock infected A549 cell sample	2.225	GSE31524	[90]
5	Ethinylestradiol study 3 (90uM) / vehicle (DMSO) treated HepG2 sample	2.02	GSE51952	[93]
6	TGF-β study 5 (intermediate) / untreated A549 cell sample	2.004	GSE17708	[91]
7	Influenza virus study 3 (A/H5N2) / mock infected A549 cell sample	1.914	GSE31524	[90]
8	Azathioprine study 7 (250uM) / vehicle (DMSO) treated HepG2 sample	1.87	GSE51952	[93]
9	Influenza virus (A/H5N2) / mock infected A549 cell sample	1.813	GSE31524	[90]
10	Heat shock / LPS / untreated THP-1 cell sample	1.76	GSE9916	[94]

GENEVESTIGATOR nomenclature is used for cell-based models/conditions. The 10-top-results for two selection categories are shown; the ranking is based on the corresponding GENEVESTIGATOR-based relative similarity (Rel.Sim). Study number and reference annotating the corresponding study are indicated.

**Table 2:** The 10-top-sphingolipid/EMT signature-linked EMT-models identified by GENEVESTIGATOR

EMT_All*				
Position	Study	Rel. Sim.	Study Number	Reference
1	TGF-β study 10 / untreated A549 cell sample	2.666	GSE49644	[95]
2	TGF-β study 5 (late) / untreated A549 cell sample	1.707	GSE17708	[91]
3	TGF-β study 12 / untreated NCI-H358 cell sample	1.490	GSE49644	[95]
4	TNF-α;TGF-β study 1 (3D) / untreated A549 cell sample (3D)	1.378	GSE42373	[96]
5	TGF-β study 5 (intermediate) / untreated A549 cell sample	1.306	GSE17708	[91]
6	TNF-α;TGF-β study 1 (2D) / untreated A549 cell sample (2D)	1.212	GSE42373	[96]
7	TGF-β study 9 / untreated PANC-1 cell sample	1.177	GSE23952	[97]
8	TNF-α; TGF-β2 study 1 (intermediate) / untreated ARPE-19 cell sample	1.170	GSE12548	[98]
9	TNF-α; TGF-β2 study 1 (early) / untreated ARPE-19 cell sample	1.152	GSE12548	[98]
10	TGF-β study 11 / untreated HCC827 cell sample	1.051	GSE49644	[95]

The 10-top-results are shown; the ranking is based on the corresponding GENEVESTIGATOR-based relative similarity (Rel.Sim). Study number and reference annotating the corresponding study are indicated.

* Selection of arrays included in this analysis is based on the knowledge-driven selection of EMT-attributed studies and is not part of a predefined GENEVESTIGATOR category.

**Table 3:** The 10-top-sphingolipid/EMT signature-linked neoplasms identified by GENEVESTIGATOR

Neoplasms_Neoplasms of Respiratory System/Intrathoracic Organs_Bronchus/Lung				
Position	Study	Rel. Sim.	Study Number	Reference
1	Adenocarcinoma study 6 (K-RAS mut) / adenocarcinoma study 6 (EML4-ALK)	2.076	GSE31210	[59]
2	Adenocarcinoma study 6 (K-RAS mut) / adenocarcinoma study 6 (EGFR mut)	1.736	GSE31210	[59]
3	Adenocarcinoma study 6 (K-RAS mut; stage II) / adenocarcinoma study 5 (K-RAS mut; stage I)	1.68	GSE31210	[59]
4	Adenocarcinoma study 6 (EGFR/K-RAS/ALK-; stage II) / adenocarcinoma study 5 (EGFR/K-RAS/ALK -; stage I)	1.476	GSE31210	[59]
5	Adenocarcinoma study 6 (EGFR mut) / adenocarcinoma study 6 (EML4-ALK)	1.267	GSE31210	[59]
6	Smoking study 13 (former smoker) / adenocarcinoma lung tissue (never smoker)	1.182	GSE12667	[99]
7	Smoking study 13 (current smoker) / adenocarcinoma lung tissue (never smoker)	0.974	GSE12667	[99]
8	Adenocarcinoma study 6 (K-RAS mut) / adenocarcinoma study 6 (EGFR/K-RAS/ALK -)	0.945	GSE31210	[59]
9	Adenocarcinoma study 5 (EML4-ALK) / adenocarcinoma study 5 (EGFR/K-RAS/ALK -)	0.94	GSE31210	[59]
10	Adenocarcinoma study 6 (EGFR mut) / adenocarcinoma study 5 (EGFR mut)	0.923	GSE31210	[59]
**Neoplasms_All**				
**Position**	**Study**	**Rel. Sim.**	**Study Number**	**Reference**
1	Adenocarcinoma study 6 (K-RAS mut) / adenocarcinoma study 6 (EML4-ALK)	2.159	GSE31210	[59]
2	Adenocarcinoma study 6 (K-RAS mut) / adenocarcinoma study 6 (EGFR mut)	1.805	GSE31210	[59]
3	Bladder cancer study 3 / bladder cancer study 2	1.803	GSE7476	[100]
4	Adenocarcinoma study 6 (K-RAS mut; stage II) / adenocarcinoma study 5 (K-RAS mut; stage I)	1.747	GSE31210	[59]
5	Colorectal cancer study 5 (crc) / colorectal cancer study 5 (vad)	1.537	GSE4183	[101]
6	Adenocarcinoma study 6 (EGFR/K-RAS/ALK-) / adenocarcinoma study 5 (EGFR/K-RAS/ALK -)	1.535	GSE31210	[59]
7	Prostate cancer study - Vegetable diet 3 (late) / vegetable diet (baseline)	1.53	E-MEX-1243	[102]
8	Prostate cancer study 6 (met. crpc) / prostate cancer study 6 (crpc)	1.509	GSE28403	[103]
9	Prostate cancer study - Vegetable diet 2 (late) / vegetable diet (baseline)	1.458	E-MEX-1243	[102]
10	Prostate cancer study - Vegetable diet 2 (early) / vegetable diet (baseline)	1.375	E-MEX-1243	[102]

GENEVESTIGATOR nomenclature is used for malignant disorders named *neoplasms*. The 10-top-results for two selection categories are shown; the ranking is based on the corresponding GENEVESTIGATOR-based relative similarity (Rel.Sim). Study number and reference annotating the corresponding study are indicated.

**Table 4:** The 10-top-sphingolipid/EMT signature-linked non-malignant conditions identified by GENEVESTIGATOR

Anatomy_Primary Cells_Respiratory System Cell_All				
Position	Study	Rel. Sim.	Study Number	Reference
1	Asthma study 1 / mock infected bronchial epithelial cell sample	1.900	GSE13396	[104]
2	TNF-α study 9 (1ng/ml) / vehicle (HBSS) treated bronchial epithelial cell sample	1.788	E-MTAB-1027	[105]
3	Asthma study 2 (human rhinovirus) / human rhinovirus study 2	1.757	GSE13396	[104]
4	TNF-α study 9 (10ng/ml) / vehicle (HBSS) treated bronchial epithelial cell sample	1.740	E-MTAB-1027	[105]
5	TNF-α study 9 (100ng/ml) / vehicle (HBSS) treated bronchial epithelial cell sample	1.698	E-MTAB-1027	[105]
6	PD-0332991 study 1 (G1 arrest) / medium treated bronchial epithelial cell sample	1.690	E-MTAB-1272	[106]
7	TNF-α study 15 (100ng/ml) / vehicle (HBSS) treated bronchial epithelial cell sample	1.670	E-MTAB-1312	[105, 106]
8	Idiopathic pulmonary fibrosis study 10 (contractile) / normal lung myofibroblast sample	1.636	GSE11196	[107]
9	TNF-α study 15 (10ng/ml) / vehicle (HBSS) treated bronchial epithelial cell sample	1.575	E-MTAB-1312	[105, 106]
10	PD-0332991 study 4 (cell cycle re-entry) / PD-0332991 study 4 (G1 arrest)	1.564	E-MTAB-1272	[106]
**Anatomy_Tissues_Organ System_Visceral Organ_Respiratory System_All**				
**Position**	**Study**	**Rel. Sim.**	**Study Number**	**Reference**
1	Chronic obstructive pulmonary disease study 10 / adjacent lung tissue sample	2.617	GSE8581	[108]
2	Lung transplantation study 1 (post-transplant) / lung transplantation study 1 (pre-transplant)	1.682	GSE18995	[109]
3	Lung transplantation study 2 (pre-transplant) / lung transplantation study 1 (pre-transplant)	1.649	GSE18995	[109]
4	Chronic obstructive pulmonary disease study 11 / adjacent lung tissue sample	1.537	GSE8581	[108]
5	Chronic obstructive pulmonary disease study 15 / normal lung sample	1.350	GSE37768	*
6	Lung transplantation study 2 (post-transplant) / lung transplantation study 2 (pre-transplant)	1.346	GSE18995	[109]
7	Smoking study 64 / normal lung sample	1.291	GSE37768	*
8	Idiopathic pulmonary fibrosis study 4 (late) / idiopathic pulmonary fibrosis study 4 (early)	1.106	GSE24206	[110]
9	Sarcoidosis study 2 / normal lung tissue	0.857	GSE16538	[111]
10	Chronic obstructive pulmonary disease study 15 / smoking study 64	0.804	GSE37768	*

GENEVESTIGATOR nomenclature is used for non-malignant conditions attributed to the respiratory system. The 10-top-results for two selection categories are shown; the ranking is based on the corresponding GENEVESTIGATOR-based relative similarity (Rel.Sim). Study number and reference annotating the corresponding study are indicated.

* Accession “GSE37768” is currently private and is scheduled to be released on Aug 25, 2016.

Applying the comparison condition platform with filter “Cell Lines_Pathological Cell Lines_Neoplastic Cell Lines_A549” (Table 1), we identified several A549 cell-based studies with similarities in gene expression profiles. Among the top 10 most significant are (i) influenza virus-infected A549 cells (human influenza virus pH1N1 and low pathogenic avian influenza viruses subtypes H5N2 and H9N2; various time points post infection; positions 1, 3, 5-10) and (ii) A549 cells treated with porcine TGFbeta (positions 2 and 4). Considering the latter finding, we additionally examined the TGFbeta-attributed sphingolipid/EMT signature and thereby identified the same A549-based study being ranked at first position (relative similarity 3.528); this outcome strongly supports the herein results and demonstrates the relevance and the power of applied strategy.

Expanding the filter to “Cell Lines_Pathological Cell Lines_Neoplastic Cell Lines_All” (Table 1), which includes additional neoplastic cell-based models, we further identified, in addition to the A549 cell-based studies described above, similarities to the transcriptional response of (iii) human pharyngeal epithelial Detroit 562 cells upon infection with Streptococcus pneumoniae (position 3); (iv) HepG2 cells treated with ethinylestradiol or azathioprine (positions 5 and 8, respectively); and of (v) THP-1 cells subjected to heat shock or lipopolysaccharide (position 10).

Additionally, we applied the filter “EMT_All” (Table 2), which includes exclusively the cell-based models of the EMT process. Although the highest similarly was observed for A549 cell-based studies (positions 1 and 2), besides we identified similarities to the transcriptional profiles of (i) other human epithelial NSCLC cell lines, such as NCI-H358 and HCC827, upon treatment with TGFbeta (position 3 and 10, respectively); (ii) human pancreatic cancer cell line PANC-1 upon treatment with TGFbeta (position 7); and of (iii) human retinal pigment epithelial cell line ARPE-19 upon treatment with TNFalpha and TGFbeta2 (position 8, 9).

We next expanded the sphingolipid/EMT-associated signature analysis from the cell-based studies to studies covering human neoplastic tissues in the bronchus and lung named “Neoplasms_Neoplasms of Respiratory System/Intrathoracic Organs_Bronchus/Lung” (Table 3). Of particular importance, the system reported similar expression patterns for primary tumor samples from patients with lung adenocarcinoma (positions 1-10) with ranking attributed to the mutation pattern either in *EGFR*, *KRAS*, or *ALK* genes. Among the triple-negative adenocarcinomas (those without EGFR, KRAS mutations and ALK rearrangements), a higher level of similarity was observed for patient group with stage II cases when compared to those with stage I (position 4).

We then asked whether the herein identified sphingolipid/EMT-associated gene signature is specific for lung cancer or can be detected in other malignant disorders. The enlarged analysis upgraded with various types of neoplasms designated herein as “Neoplasms_All” (Table 3) identified the highest similarity to expression patterns of (i) as above, lung adenocarcinoma (positions 1, 2, 4, and 6); (ii) bladder cancer (position 3); (iii) colorectal cancer (position 5); and (iv) prostate cancer (positions 7-10). In sum, the outcome indicate that the A549 cell-based sphingolipid/EMT-related signature composed of genes differentially expressed at the EMT stage is closely related to the one identified in specimens of patients with lung adenocarcimoma, thus strongly supporting the clinicopathological relevance. Notably, this signature also showed similarity to the expression profiles in some, though few, other types of solid tumors.

We additionally extended the scope of analysis to non-malignant settings attributed to the respiratory system including the cell-based models/treatment conditions of primary cells and non-malignant conditions at the organ system level. The comparison condition platform for primary human cells with the filter “Anatomy_Primary Cells_Respiratory System Cell_All” (Table 4) identified similar expression patterns in (i) primary bronchial epithelial cells from patients with mild asthma (positions 1 and 3); (ii) normal bronchial epithelial cells upon treatment with TNFalpha or PD-0332991 (positions 2, 4, 5, 7, 9 and 6, 10, respectively); and in primary lung myofibroblasts expanded from patients with idiopathic pulmonary fibrosis (position 8). Applying the comparison condition platform with the filter “Anatomy_Tissues_Organ System_Visceral Organ_Respiratory System_All” (Table 4), we identified the highest similarity to expression patterns observed in studies attributed to (i) the chronic obstructive pulmonary disease (positions 1, 4, 5, and 10); (ii) lung transplantation conditions (positions 2, 3, and 6); (iii) cigarette smoke (position 7 and 10); (iv) idiopathic pulmonary fibrosis (position 8); and (v) pulmonary sarcoidosis (position 9).

## DISCUSSION

To study interrelations between the sphingolipid machinery and the EMT program in lung cancer and accounting for complexities of both biological systems, in this work we applied an integrative strategy, which covers the levels of the biological hierarchy from molecules to functional gene network(s) and biological pathways and further to pathophysiological disease conditions/situations. Our underlying experimental methodology is based on the biological process-focused profiling approach, using the expert-designed gene panels of the most relevant genes (biology- and knowledge-driven) and the well-characterized *in vitro* cell-based models, followed by the mining of multiple knowledge-based data sets from variety of experimental platforms and reconstruction of associated networks and biological mechanisms in the context of disease pathobiology. This study highlights novel sphingolipid-associated mechanisms underlying the EMT and provides a comprehensive overview of sphingolipid/EMT-related biological aspects with strong indication that the herein dissected sphingolipid/EMT-associated mechanisms may impact — among the heterogeneous NSCLC types — the pathobiology of lung adenocarcinoma.

Our first contribution shows that the sphingolipid/EMT-associated signature is able to differentiate the epithelial stage-lung cancer cells versus those undergone EMT. This study grouped the EMT-regulated genes with S1P- and Cer-modulating enzymes, S1P and LPA receptors as well as S1P transporters. It helped us to discriminate whether the particular gene is responsive either to TGFbeta and/or TNFalpha, or shows additive or synergist response to the combination of stimuli, which in turn mimics the pro-inflammatory milieu of tumor microenvironment. Notably, the herein identified sphingolipid/EMT-associated differential gene expression pattern was accompanied by the significant decrease in cellular Cer paralleled by increase in cellular S1P in A549 cells that have undergone an EMT. This shift in bioactive sphingolipid mediators is strongly in favour of promoting the cell survival and enhancing the invasion properties, thus, emphasizing the role of sphingolipid-related mechanisms in EMT program. Profiling in respect of S1P-modulating enzymes revealed, on one side, strong upregulation of S1P-producing *SPHK1*, while, on the other side, strong upregulation of S1P phosphatases, *SGPP2* and *PPAP2B*, as well as moderate upregulation of S1P-degrading *SGPL1*. Thus, the SPHK1-driven effect(s) seems to play a dominant role in respect of S1P metabolism in A549-based EMT process triggered by TGFbeta + TNFalpha and results in accumulation of intracellular S1P at the mesenchymal/fibroblast-like stage. These results are strongly supportive and additive to the recent data of Milara et al. [29] showing upregulation of SPHK1 on the protein level in TGFbeta-triggered A549 cell-based EMT; thus, SPHK1/S1P axis is important in TGFbeta signaling, whereas TNFalpha potentiates the TGFbeta-triggered SPHK1 expression/activity. Enhanced PPAP2B in turn, which is known to act as S1P ecto-phosphatase, might impair the S1P-mediated reactivation of tumor cells in autocrine mode via S1PRs and redirect its action to the paracrine manner; approximation of these findings to disease conditions suggests that the S1P effects on stroma and/or immune cells composing the tumor microenvironment thereby might be enhanced. This hypothesis is strengthened by recent data of Albinet et al. [18], who defined principal role of SPHK1/S1P in melanoma-stroma interactions by showing that SPHK1-active melanoma cells triggered the differentiation of fibroblasts to myofibroblasts and increased the production of matrix metalloproteinases. We speculate that PPAP2B-driven decrease of extracellular S1P levels in close proximity to the tumor cell at the EMT stage might build up an S1P gradient facilitating the motility of this subpopulation of tumor cells. Importantly, considering the broad specificity of PPAP2B against phosphorylated lipid mediators [30, 31] the same mechanism(s) might be relevant for LPA and C1P during EMT process. In support, recent study by Muinonen-Martin et al. [32] demonstrated the outward-facing gradient of LPA *in vitro* and, moreover, *in vivo* across the margins of melanomas thereby emphasizing the pathophysiological relevance of the LPA-mediated chemotaxis as one of the strongest driving factor for melanoma cell invasion. Thus, our results suggest that both S1P- and LPA-coupled biological functions contribute during the EMT program and emphasize the participating roles of both S1P and LPA receptors. We describe a novel role of TGFbeta in the control of LPAR5 expression and the potential LPAR5-attributed biological mechanisms, based on mRNA expression analysis. *LPAR1* in contrast showed a repressed expression in the presence of TGFbeta in line with recent findings of Wu et al. [33]. Considering that generally very limited information is currently available about LPARs regulation on transcriptional levels, our data not only provide an overview of LPAR repertoires characteristic for epithelial versus mesenchymal/fibroblast-like cells, but also align the LPARs and S1PRs expression patterns and thus offer potential targeting strategies, including the option of combinatorial targeting of receptor(s) for both S1P and LPA.

As novel evidence of potential contribution of LPA to the EMT process, herein we show that the mesenchymal/fibroblast-like stage was characterized by the significant increase in intracellular LPA levels; to the best of our knowledge, this is the first study addressing the EMT-associated alterations of this bioactive lysophospholipid in tumor cells. Intracellular LPA production involves phospholipase enzymes [34, 35], not autotaxin (which in turn regulates extracellular LPA production in biological fluids), and is generally less studied. If to consider that *ENPP2* (also known as *LysoPLD* or *ATX*) encoding autotaxin is not expressed by A549 cells in the herein EMT model (gene expression data), primarily the increased intracellular LPA pool might have functional consequences contributing to EMT in lung cancer cells.

The data furthermore suggest that besides the S1P and LPA axes, the ceramide-coupled events might play a contributing role to the EMT program. One interesting aspect is based on the potential linkage between cellular ceramide content, membrane fluidity and cell motility. The EMT-driven altered expression of any Cer-producing or Cer-utilizing enzymes, and thus modulation of the ceramide pool, might contribute to those cellular events. In support, recent findings indicate that downregulation of CERS6 in breast cancer cells *in vitro* resulted in enhanced membrane fluidity and stimulated cell migration [36]. Intriguingly, we observe the EMT-driven moderate upregulation of Cer-utilizing *CERK*. The ceramide-sphingomyelin enzymatic branch shows minor regulations being reflected by unmodulated sphingomyelin levels. Another prominent ceramide function is given by a well-known role of Cer molecule in the regulation of diverse types of apoptosis [37, 38]; thus, the herein Cer-attributed data corroborate the pro-survival phenotype of cells that have undergone EMT [39]. In sum, our data indicate that the EMT stage is characterized by reduced cellular Cer levels and thereby propose a novel biological link between ceramide and the metastatic potential of lung cancer cells. In respect of HexCer, the herein observed decrease in lipid level might be attributed to decrease of Cer as lipid precursor; still, we cannot exclude that the significant decrease in HexCer has as well functional consequences in the EMT process.

We also show EMT-associated changes in the levels of S1P transporters. Recent studies attract particular attention to the efflux S1P transporters and nominate them as promising targets for cancer therapy (recently reviewed in [40]). Noteworthy, for patients with lung adenocarcinoma a potential link between the ABCG2 expression and clinical outcome has been proposed; thus, expression of ABCG2 in combination with CD133 expression (ABCG2^+^/CD133^+^ NSCLC status) was found to be a predictor of a high risk of early relapse [41]. Additionally, the single nucleotide polymorphism within ABCG2 was shown to be associated with the clinical outcome in patients with unresectable NSCLC patients treated with the first-line platinum-based chemotherapy [42]. Moreover, ABCG2 is a potential target causing the multidrug resistance in NSCLC; suppression of *ABCG2* transcription, as shown in A549 cell-based models [43, 44], may reverse the multidrug resistance and thus enhance the effectiveness of other anti-cancer drugs. Considering such multifactorial contribution of ABCG2 in lung cancer pathogenesis, the novel finding of the current study associating the modulation of ABCG2 expression within the EMT program and the S1P biology is of particular interest. We herein show that *ABCG2* mRNA expression levels are strongly downregulated at EMT triggered by both TGFbeta, TNFalpha and even more pronounced by their combination. Notably, such expression pattern shows the closest association with the one of the classical EMT marker, *CDH1*/*E-cadherin*. These findings propose to consider the multifactorial ABCG2 molecule as novel EMT marker and to further study its functional relevance for EMT program in lung cancer in relation to the sphingolipid-associated mechanisms and the multidrug resistance regulation as assessed in [43, 44]. In respect of more specific S1P transporters, we herein show that *SPNS2* is attributed to a low expressing gene in A549 cells at epithelial stage and its transcription can be transiently stimulated by the combination of TGFbeta and TNFalpha with a maximum mRNA expression detected at 2.5 h time point. However, at the mesenchymal stage *SPNS2* mRNA levels are downregulated even below the basal level. Given the recent findings indicating that the plasmid-based overexpression of SPNS2 in A549 cells induces apoptosis [45], the herein presented data suggest that the EMT-associated downregulation of *SPNS2* expression may contribute to the survival phenotype of A549 cells at the mesenchymal stage. It is interesting to note that siRNA-mediated SPNS2 knockdown in A549 cells was accompanied by the significant increase in intracellular S1P levels and led to enhanced cell migration [45], thus being in line with the herein described characteristics of A549 cells at the mesenchymal/fibroblast-like stage. In sum, the findings suggest that modulated expression of SPNS2 is one of the components within the complex network of sphingolipid machinery contributing to EMT program in NSCLC.

Recent evidence suggests that interaction of tissue infiltrating immune cells with an inflammatory phenotype with epithelial cells accelerates the TGFbeta-triggered EMT process in part via the interaction of LFA-1 on leukocytes and ICAM1 expressed on epithelial cells [46, 47]. Moreover, recent findings demonstrate that S1P is able to enhance ICAM1 expression on human pulmonary alveolar epithelial cells and thus impact on ICAM1-dependent monocyte adhesion to epithelial cells [48]. In this regard it is important to note that in our study the EMT phenotype in A549 cells was accompanied by strong enhancement of ICAM1 expression and increased cellular S1P levels. These cross-talks may lead to an amplifying loop between S1P, TGFbeta and ICAM1 thereby contributing to multiple cellular activities. In the current study we showed the EMT-upregulated expression of CD40 on mRNA and protein levels. Noteworthy, enhanced CD40 expression in NSCLC was shown to correlate with poor prognosis [49]. Of note, unsupervised clustering across all stimulation conditions revealed close association between *CD40*, *ICAM1*, *TNF* and pluripotency markers; furthermore, close associations within the same cluster were shown with genes of sphingolipid machinery including *PPAP2B* and *SGPP2*, suggesting that these lipid phosphatases can manipulate inflammatory responses and alter an EMT-associated pathomechanisms of diseases with inflammatory background as proposed by us previously [7, 30].

Overall, these results points out that the sphingolipid-associated mechanism(s) can influence several aspects of the EMT program. To gain insight into underlying cellular mechanisms, we analysed our own data in the context of previous knowledge and for that linked the signature-derived information to the known Canonical Pathways and Upstream Regulators (Figure 8). We identified, besides the presumably expected sphingolipid/LPA-driven mechanisms and the EMT program, as well as anticipated stemness/pluripotency mechanisms, additional interconnections with fibrosis, inflammation/immune-related mechanisms and the integrin-mediated processes. Importantly, with respect to the ILK signaling, recent data emphasize the critical role of ILK in EMT [50] and, moreover, highlight its clinical relevance for patients with NSCLC [51]. However, it is — to our knowledge — the first demonstration of potential interconnection of ILK-mediated events with the sphingolipid machinery. This is as well relevant for some candidate molecules among the herein identified Upstream Regulators. Strikingly, the transcriptional regulator SPDEF was recently shown to act as a tumor metastasis suppressor *in vivo* [52] and the multifactorial adaptor protein MAD2L2 (also known as MAD2B) was proposed to be involved in the TCF4-mediated epithelial-mesenchymal transdifferentiation [53]. Recent evidences indicate a cross-talk between TGF-β- and Wnt-associated pathways and a developmental pathway called Hippo; it is thereby proposed that targeting Hippo signaling might be an effective strategy in inhibiting EMT [54]. In respect of sphingolipid signaling, modulation of Cer levels can affect the Wnt signaling [55], while S1P and LPA might modulate the components of Hippo pathway [56]. In the context of herein described results, the Wnt/beta-catenin signaling pathway is among the significant Canonical Pathways and its signaling components are among the Upstream Regulators, although outside the 10 top outcomes.

Thus, in sum our work has led to the nomination of new sphingolipid/EMT-associated biologically related pathways and regulatory molecules and identified future research directions. Follow-up studies will prove on the protein level the gene signature-derived alterations and define the functional effects of the candidate molecules on the EMT program triggered in lung cancer cells.

In order to answer questions about the specificity of our signature and its relevance for diseased settings, the signature was compared to data sets from manually curated microarray-based studies. Among the 10 top outcomes, we indeed detected the indicated sphingolipid/EMT signature in A549 cell-based model upon treatment with TGFbeta; this highlights the reproducibility of our findings between different laboratories and, strikingly, at least for the indicated set of genes, between different methodologies (the real-time PCR-based profiling versus microarray analysis) and thus supports the relevance and the power of the applied approach. Rather unexpectedly, our data link the sphingolipid/EMT signature with the transcriptional A549 cell state attributed to infection with human influenza virus strain (pH1N1) and low pathogenic avian influenza viruses (H5N2 and H9N2). Although it is well established that viruses generally exploit membranes composing of sphingolipids in multiple steps of their cycle and are known modulators of the sphingolipid metabolism as well as signaling pathways (recently reviewed in [57, 58]), this finding — to the best of our knowledge — for the first time suggests a cross-talk between the sphingolipid/EMT-related mechanisms and the influenza virus-host interactions on the level of epithelial cells. Extending the comparison conditions to the wide variety of (i) neoplastic cell-based models and (ii) cell-based EMT models, firstly, confirmed the strongest association with the A549 cell-based studies and, secondly, revealed similarities to the transcriptional responses triggered in other types of neoplastic cell lines and other cell-based EMT models of various origins indicating that the defined signature is highly specific but not exclusive for the EMT process in lung cancer A549 cells. Intriguingly, under certain circumstances, this transcriptional switch might occur in primary bronchial epithelial cells and lung myofibroblasts.

We next showed that our signature is furthermore relevant for diseased conditions. It is important to note that with respect to lung cancer — a highly heterogeneous disease subdivided in various types differing in disease patterns and treatment strategies [27] — the closest association was found with adenocarcinoma, which is the most common type of NSCLC. The study assessing molecular characteristics of lung adenocarcinomas, which harbor *EGFR, KRAS* mutations or *ALK* rearrangements as well as triple negative adenocarcinomas, dominated the top 10 alignment outcomes [59]. Noteworthy, the comparison in expression profiles between the stage II adenocarcinomas carrying *KRAS* mutations or *EML4*-*ALK* fusions was ranked to position 1. Since other comparisons in respect of mutational status of adenocarcinomas has been as well selected within the top 10 output results, we cannot make a definite conclusion whether the sphingolipid/EMT signature is able to differentiate the particular mutational status or underlines the critical pathomechanism(s) of lung adenocarcinomas in general. Taken together, these data emphasize the relevance of the A549 cell-derived sphingolipid/EMT signature for patients with lung adenocarcinoma and thus strongly propose the impact of sphingolipid/EMT-associated events to complex disease biology of NSCLC. Given the emerging role of pathway-relevant signatures as basis for prognostic/predictive models linking the disease-associated gene alterations with the clinical outcome in a patient-orientated manner ([60–62] and our recent study in terms of the AID/APOBEC signature in ovarian cancer; submitted), it should be rational and achievable to apply the herein defined sphingolipid/EMT signature for assessment of NSCLC patient risk/survival. In this context, it is worth investigating whether an optimization of a signature composition still may improve the performance for prognostic or predictive models [63].

Finally, we assessed whether the herein defined signature is unique for the lung cancer or is common for other types of malignant disorders. The extended transcriptome-based analysis across diverse types of neoplasms ranked, as before, the lung adenocarcinoma-associated profiles at first two positions. Besides the lung adenocarcinoma, among the top 10 neoplasms the closest associations were found for bladder, colorectal and prostate cancer. Several distinct mechanisms have been nominated to trigger the EMT program in bladder cancer, which were shown to contribute to cancer progression by regulating the drug resistance and invasion/metastasis [64–67]; in colorectal cancer the EMT process has been as well associated with disease progression and poor survival prognosis, while the inverse MET stage was characteristic for established metastases in the liver [68–72]; in prostate cancer the EMT was suggested to contribute to osteoblastic bone metastases [73–75]. Yet — to the best of our knowledge — in none of these cancer types the direct link between EMT process and the sphingolipid machinery has been shown.

## MATERIALS AND METHODS

### Cell culture and cell stimulation

The lung adenocarcinoma epithelial cell line, A549, was obtained from the American Type Culture Collection (Manassas, VA, USA) and kindly provided by Prof. Gerhard Hamilton; cells were maintained in RPMI 1640 (PAN™ Biotech) supplemented with 10% fetal bovine serum (FBS, Gibco by Life Technologies) at 37°C, 5% CO_2_. Cell stimulation was performed in RPMI 1640 (PAN™ Biotech) supplemented with 5% FBS (Gibco by Life Technologies). To trigger the EMT program, cells were seeded in 6-well plates (for RNA isolation and flow cytometry) or in T75 flasks (for lipid extraction) to achieve 40% confluency on the next day and stimulated with TGFbeta at 2 ng/ml (TGF-β1, R&D Systems), TNFalpha at 12.5 ng/ml (human TNF-α, PeproTech) or their combination for 1 h, 2.5 h, 6 h, 24 h, and 48 h. Protocol included a re-stimulation step at 24 h time point. Stimulation conditions for triggering EMT in A549 cells were described previously [28].

### Microscopic evaluation, flow cytometry and immunofluorescence analysis

A549-based EMT model was triggered by TGFbeta, TNFalpha or their combination for 48 h. Epithelial versus mesenchymal/fibroblast-like characteristics were assessed microscopically and by flow cytometry. Microscopic images were acquired in the bright-field mode using an inverted microscope Axio Observer Z1 equipped with a high resolution AxioCam MRc 5 camera (Carl Zeiss). For flow cytometric analysis, cells were stained at 4°C for 30 minutes with the following antibodies: mouse anti-human CD324 (E-Cadherin)-Alexa Fluor 647 (BD Pharmigen), mouse anti-human CD90-FITC (Acris Antibodies GmbH), mouse anti-human CD40-R-PE (Invitrogen), mouse anti-human CD54 (ICAM1)-PE (BD Pharmigen). Samples were analyzed by BD FACSCanto II (BD Biosciences); data were analyzed using FlowJo software (BD Biosciences). Immunofluorescence microscopy was performed according to the protocol previously established by us [7, 76]. Briefly, A549 cells were grown overnight on Permanox 8-well chamber slides (Nunc). Cells were stimulated with TGFbeta + TNFalpha for 48 h or left untreated. Upon stimulation, cells were fixed with 3.7% formaldehyde and permeabilized with 0.5% Triton X-100 in PBS. Primary antibodies against vimentin (Cell Signaling) were diluted in PBS, 0.5% bovine serum albumin and incubated with the cells for 1 h at room temperature. Upon washing, cells were incubated with goat anti-rabbit antibodies labelled with Alexa 488 (Invitrogen) for 45 minutes at room temperature. Nuclei were stained with DAPI (Roche). Slides were scanned using microscopy-based TissueFAXS platform (TissueGnostics, Vienna, Austria) equipped with Hamamatsu ORCA flash 4.0 camera (Japan) and Plan-Apochromat 63x/1.4 Oil objective (Zeiss). Filter sets were from Chroma TechnologyCorp (DAPI 350/460 nm, FITC/Cy2 470/525 nm).

### Real-time PCR analysis

A549 cells were stimulated in kinetics or left untreated as described above; total RNA was isolated using peq GOLD TriFAST™ (Peqlab) reagent according to manufacturer's protocol. 1 μg RNA was used for cDNA generation using the High Capacity cDNA Reverse Transcription Kit (Applied Biosystems by Life Technologies) according to the instructions of the manufacturer. Real-time PCR analysis was performed using Power SYBR Green PCR Master Mix (Applied Biosystems by Life Technologies) in the 384-well plate format on ABI 7900HT instrument equipped with SDS 2.3 software (Applied Biosystems by Life Technologies). All primers were self-designed using the Primer Express 3.0 software (Applied Biosystems by Life Technologies) and validated using the Human Total RNA Master Panel (Takara, Clontech Laboratories Inc.,) as described [7, 62]. Primer sequences are summarized in Supplementary Table S5. *EF1A* was used as housekeeping gene (HKG) for normalization as assessed using the DataAssist (Applied Biosystems)-based selection for the most stable expression along the treatment conditions among three pre-tested HKGs (*EF1A*, *UBC*, and *HPRT*). For relative quantification, data were analyzed by ΔΔCT method. Expression levels of target genes were normalized to HKG and shown relative to unstimulated cells (0 h time point). Expression profiling was performed using the EMT-associated and sphingolipid-associated multigene signatures; the compositions of these self-created signatures are described below.

### The EMT-associated multigene signature used for characterization of A549 cell-based EMT model

To monitor the entire EMT process on the mRNA level we created an EMT-associated signature covering the key players of the transition program; the composition was assembled based on a knowledge-driven approach as described previously [62]. The gene signature includes (i) the cell adhesion molecules *CDH1* (also known as *E-Cadherin*) and *CDH2* (also known as *N-Cadherin*); (ii) the transcriptional regulators of the *SNAI*, *ZEB* and *TWIST* families; (iii) the fibroblast marker *THY1* (also known as *CD90*); (iv) the cytoskeleton components *VIM*, *ACTA2* and *S100A4*. Additionally, (v) the cell plasticity/pluripotency markers such as *NANOG*, *DPPA3* (also known as *Stella*), *CD34*, and *PROM1* as well as (vi) inflammation-associated molecules *CD40* and *ICAM1* (also known as *CD54*) were included. Furthermore, we enlarged the signature by (vii) the known TNFalpha-triggered, NFkappaB-dependent target genes, *TNF* and *IL6,* as well as the TGFbeta-triggered target genes, *SERPINE1* (also known as *PAI1*) and *CTGF* to confirm the stimulatory effect of both cytokines. In sum, the EMT-associated signature includes 23 genes. Gene symbol, synonyms, gene name, NCBI accession number, and short functional description are provided in Supplementary Table S6.

### The composition of the sphingolipid-related multigene signature

To assess a role of sphingolipid machinery in the EMT process, we created a sphingolipid-associated multigene signature (n=41); given a cross-talk between S1P and LPA in respect of some metabolizing enzymes and downstream signaling, the signature was upgraded by LPA receptors. The multigene signature thus covers (i) the interconnected gene network of the sphingomyelin/salvage pathway including genes encoding the S1P-modifying enzymes such as two families of phosphatases *SGPP1* and *SGPP2*, and *PPAP2A*, *PPAP2C*, *PPAP2B* (also known as *LPP1*, *LPP2* and *LPP3*, respectively; in addition to S1P, those phosphatases are known to dephosphorylate PA, LPA and C1P [77], the S1P-degrading *SGPL1* and the S1P-producing sphingosine kinases *SPHK1* and *SPHK2*; phospholipase *ENPP2* (also known as *LysoPLD*); genes encoding the Cer-modifying enzymes including Cer-producing *SMPD1* (also known as *ASMASE*), *SMPD2*, *SMPD3*, *SMPD4* (also known as *NSMASE1*, *NSMASE2*, *NSMASE3*, respectively), and family of Cer synthases *CERS1*, *CERS2*, *CERS3*, *CERS4*, *CERS5*, and *CERS6*; the ceramidases *ASAH1* and *NAAA* (also known as *ASAHL*); the C1P-producing ceramide kinase *CERK*; SM-producing *SGMS1* and *SGMS2*; transferases utilizing Cer *UGT8* and *UGCG*; (ii) the family of G-protein coupled S1P receptors, *S1PR1*, *S1PR2*, *S1PR3*, *S1PR4*, and *S1PR5*, and the related family of LPA receptors, *LPAR1*, *LPAR2*, *LPAR3*, *LPAR4*, *LPAR5*, and *LPAR6*; (iii) potential S1P transporters of ABC family, *ABCA1*, *ABCC1*, *ABCG2*, and *SPNS2* (reviewed in [40]). The signature does not cover genes encoding the enzymatic machinery for *de novo* sphingolipid biosynthesis, ACER family, and genes encoding the enzymes of complex glycosphingolipids biosynthesis. Gene symbol, synonyms, gene name, NCBI accession number, and short functional description of genes composing the signature are provided in Supplementary Table S6.

### Lipid extraction and mass spectrometric analysis

For Cer, HexCer and SM lipid extraction was performed according to Matyash et al. [78]. Briefly, cell pellets were homogenized in a mixture of 1.5 ml MeOH and 3 ml methyl tert-butyl ether (MTBE) and 1.25 ml aqua bidest was added thereafter. After vigorous shaking the upper layer was taken off and the remaining solvent was re-extracted with a mixture of MTBE/MetOH /aqua bidest (10/3/2.5 per vol.). The pooled extracts were evaporated in a SpeedVac. For mild alkaline hydrolysis, 400 μl of CHCl_3_/MeOH/H_2_O (16/16/5 per vol.) were added to the solvent-free lipid extracts and the solution was shaken vigorously. After addition of 400 μl 0.2 M NaOH in MeOH, the samples were incubated at room temperature for 45 minutes. Following addition of 400 μl 0.5 M EDTA and 150 μl CH_3_COOH and vigorous shaking, 1 ml CHCl_3_ was added to extract the lipids. Extracts were shaken for 5 minutes and centrifuged for 3 minutes at 300 g to facilitate phase separation. The chloroform phase was transferred to a new vial and the solvent was removed under a nitrogen stream. Dried lipid extracts were resuspended in 100 μl CHCl_3_/MeOH (1/1, v/v) containing 15 pmol Cer 12:0, Cer 25:0, HexCer 12:0, and SM 12:0 each as internal standards. Chromatographic separation of lipids was performed by an Accela HPLC (Thermo Scientific) on a Thermo Hypersil GOLD C18, 100 × 1 mm, 1.9 μm column. Solvent A was a water solution of 1% ammonium acetate (v/v) and 0.1% formic acid (v/v) and solvent B was acetonitrile/2-propanol (5/2, v/v) supplemented with 1% ammonium acetate (v/v) and 0.1% formic acid (v/v), respectively. The gradient was run from 35% to 70% B for 4 min, then to 100% B in additional 16 min with subsequent hold at 100% for 10 min. The flow rate was 250 μl/min. Sphingolipid species were determined by a TSQ Quantum Ultra (Thermo Scientific) triple quadrupole instrument in positive ESI mode. The spray voltage was set to 5000 V and capillary voltage to 35 V. SM, HexCer and Cer species were detected in positive ionization by precursor ion scan on m/z 184 at 35 eV and on m/z 264 at 30 eV respectively as described previously [79, 80]. Peak areas were calculated by QuanBrowser for all lipid species and quantitation was done by correlation to internal standards. Data are shown as pmol per 10^6^ cells.

For S1P extraction the butanolic extraction procedure was adapted from Baker et al. [81] and Scherer et al. [82]. Cell pellet was homogenized in 400 μl of 30 mM citric acid/40 mM Na_2_HPO_4_. Samples were spiked with 5 μl of internal standard (S1P, d17:1, 95 μM in 95% MeOH, Avanti Polar Lipids). Suspension was transferred into a 10 ml glass tube and washed with 400 μl 30 mM citric acid/40 mM Na_2_HPO_4_ with vortexing. Next, 2 ml water- saturated butanol were added and sample was vortexed for 5 min followed by incubation in the ultrasonic bath for 15 minutes at full power. To facilitate phase separation, samples were centrifuged for 15 min at 1000 rpm. The upper phase was collected and evaporated in a SpeedVac (20 min at 35°C) till less than 20 μl were left. For analysis samples were filled up to 200 μl with solvent A (H_2_O/ACN/FA, 70:30:0.2, v/v), vortexed and ultra-sonicated for 5 min. Analysis was performed using an Agilent 1290 Infinity UHPLC coupled with a Thermo Q-Exactive Orbitrap. On the UHPLC, 20 μl per sample were injected and separated using reversed phase column (2.1 mm × 15 cm, 2.6 μm, C18, 100 Å, Phenomenex Kinetex™), by implementing a 250 μl/min flow rate of 30-98% of solvent B (2-Propanol/ACN/FA, 50:50:0.2, v/v) over a 10 min gradient along with solvent A. For mass spectrometric detection, MS scans were performed in negative ion mode, with a range from m/z 280 to 650 at a resolution of 35 000 (at m/z = 300). MS/MS scans of the six most abundant ions were achieved through HCD fragmentation at 30% normalized collision energy and analyzed in the Orbitrap instrument at a resolution of 17 500 (at m/z = 300). AUC (area under the curve) of both S1P (d18:1) and the internal standard (S1P, d17:1) were obtained by calculating the corresponding peak area. Next, AUC of S1P was (d18:1) normalized to AUC of internal standard (S1P, d17:1) and shown as AUC per 10^6^ cells.

For LPA species, the same extraction protocol was used as described above for S1P; LPA 17:0 and LPA 17:1 (Avanti Polar Lipids) were used as internal standards. For analysis, samples were filled up to 200 μl with solvent B (2-Propanol/ACN/MeOH/FA, 60:20:20:0.5, v/v), vortexed and ultra-sonicated for 5 min. Analysis was performed using Dionex Ulimate 3000 UHPLC coupled with a Thermo Velos Orbitrap. On the UHPLC, 20 μl per sample were injected and separated using reversed phase column (3 mm × 15 cm, Luna-C8, 100 Å, Phenomenex), by implementing a 1000 μl/min flow rate of 70-98% of solvent B over a 12 min gradient along with solvent A (H20/MeOH/FA, 90:10:0.5, v/v). For mass spectrometric detection, MS scans were performed in negative ion mode, with a range from m/z 350 to 550 at a resolution of 35 000 (at m/z = 400). MS/MS scans of the five most abundant ions were achieved through HCD fragmentation at 35% normalized collision energy and analyzed in the Orbitrap at a resolution of 7500 (at m/z = 400). AUC of both LPA species and the internal standards were obtained by calculating the corresponding peak area. Next, the individual AUCs were normalized to the average value of AUCs of internal standards and shown as AUC per 10^6^ cells.

### Statistical analysis, data interpretation and visualization

The statistical significance of the mass spectrometry-derived data was assessed with the two-tailed *t* test; p≤0.05 was considered statistically significant. For real-time PCR-based outcomes relative mRNA levels (relative to time point 0 h) were log2 transformed and then analyzed, for each gene separately, by two-way analysis of variance (ANOVA), including factors for induction time and treatment and their interaction. Mean log2 relative expressions were evaluated, using the ANOVA model, at 1 h, 2.5 h, 6 h, 24 h, and 48 h of induction under the three treatments. For each time point, we tested the null hypothesis that mean log2 relative expression is equal to zero based on the ANOVA model. Since the primary outcome parameter was the relative expression at 48 h, the corresponding p-value was not adjusted for multiple comparisons. The other p-values (1 h, 2.5 h, 6 h, 24 h) were adjusted for multiple comparisons by the Bonferroni-Holm method. All p-values were two-sided, and considered as indicating statistical significance if lower than 0.05. The SAS System Version 9.4 (2012 SAS Institute Inc., Cary, NC, USA) was used for statistical analysis. Spearman's correlation was performed for each individual gene contributing to the sphingolipid/EMT-associated 35-gene signature, across all time points and treatment conditions. Clustering analysis and follow up graphical representation was performed using Cluster 3.0 and Java TreeView programs. Pearson uncentered hierarchical clustering was applied on log2 transformed fold-change values of gene expression data sets. Correlation analysis was performed using Pearson's correlation for log2 transformed expression data sets using SPSS software version 22 (IBM Corporation) and R package fdrtool [83] for FDR correction. The correlation matrix-based heat map was created using the Spotfire software [84]; thereby clustering method with Euclidean distance measure and average value as ordering weight was applied. The data-driven, sphingolipid/EMT-associated gene network reconstruction was created using the Ingenuity Pathway Analysis (IPA, [85]) tool; the sphingolipid/EMT signature-based genes which showed regulated expression at the EMT stage (fold change ≥2 for upregulated and ≤0.5 for downregulated genes at 48 h TGFbeta+TNFalpha, Figures 3 and 4) were assigned to Canonical Pathways as well as Upstream Regulators. The corresponding IPA-derived p-value determines the probability that the association between the genes from expression profiling data set and a Canonical Pathway/ Upstream Regulator can be explained by chance alone. The top ranking was based on the p-value. The following strategy was used to depict Upstream Regulators to be used for network reconstruction. The IPA Upstream Regulator module predicted 2982 significant molecules. Filter was applied to select the molecules of type ‘Genes/RNA/Protein’ which are assigned with ‘Predicted Activation State’ including ‘Activated’ or ‘Inhibited’. Next, the top most significant were selected and upgraded with those upstream regulators that are also assessed in our experiment (TNFalpha, SERPINE1 and IL6). We additionally added S1P and LPA as IPA knowledge-based non-gene regulators of sphingolipid machinery.

### Analysis of perturbations and diseased conditions showing similarities to signature-based profile using curated public microarray data sets

For the *in silico* identification of conditions — including neoplastic cell-based systems and lung or other cancers — that show similarities in gene expression pattern to the sphingolipid/EMT signature, we used the Signature tool from the GENEVESTIGATOR search engine [86]. GENEVESTIGATOR is a web-based analysis platform for manually curated and globally normalized transcriptomic data sets [87]. For analysis, we selected data sets from the Affymetrix Human Genome U133 Plus 2.0 Array platform; out of a total of 46323 arrays, the category “Cell Lines_Pathological Cell Lines_Neoplastic Cell Lines_A549” includes 183 arrays, the category “Cell Lines_Pathological Cell Lines_Neoplastic Cell Lines_All” included 4154 arrays, the category “Neoplasms_Neoplasms of Respiratory System/Intrathoracic Organs_Bronchus/Lung” included 1440 arrays, the category “Neoplasms_All” included 19944 arrays, the category “Anatomy_Primary Cells_Respiratory System Cell_All” included 1360 arrays, the category “Anatomy_Tissues_Organ System_Visceral Organ_Respiratory System_All” included 501 arrays, and the category “EMT_All” included 95 arrays; for the latter, knowledge-driven selection of EMT-attributed microarray data sets was performed. To fulfil study-driven special requests in respect of recently released study-relevant microarray data sets the GENEVESTIGATOR platform was upgraded with additional EMT-attributed studies. Selection of eligible Affymetrix probes for the genes composing the sphingolipid/EMT signature was done using the open access Genevisible tool powered by GENEVESTIGATOR [88]. The expression pattern of the sphingolipid/EMT gene signature which showed regulated expression at the EMT stage (fold change ≥2 for upregulated and ≤0.5 for downregulated genes at 48 h TGFbeta+TNFalpha, Figures 3 and 4) was aligned using Pearson's correlation with log2 transformed expression values of microarray data sets from the categories indicated above. The result then shows conditions, which are similar to the entered genes and relative expression values. The relative similarity indicates the degree of their resemblance. More precisely, if the similarity is defined as 1/*d_i_* with *d_i_* the distance of category *i* to the signature, then the relative similarity R of a category *c* is calculated as.

$$R_{s_{c}}=\frac{s_{c}}{\frac{1}{N}\Sigma_{i\in I}s_{i}}.$$

The ranking was done on the basis of relative similarity and the top 10 most correlated conditions were selected.

## CONCLUSIONS

Our study represents to our knowledge the first integrative approach to link the sphingolipid machinery and the EMT process in lung adenocarcinoma. We developed a strategy that allows to bridge the cell-based outcomes with the disease settings and thereby validate their biological and clinical relevance in terms of underlying mechanisms contributing to disease pathogenesis. We created and successfully applied for the lung cancer cell-based EMT model the combination of multigene signatures depicting the transcriptional profile of the EMT program and covering the sphingolipid-related gene network; the resulting 35-gene sphingolipid/EMT signature is able to differentiate the epithelial cell stage and the mesenchymal/fibroblast-like cell stage with high precision. As side application, the sphingolipid/EMT-associated multigene signature can be proposed to be used as novel EMT interfering drug screening system for sphingolipid-related targets and beyond.

We demonstrate that there are many sphingolipid-related aspects of EMT biology that were unexamined till now. We identified the sphingolipid/EMT-related genes among the S1P-metabolizing enzymes, S1PRs and S1P transporters as well as LPARs; we furthermore showed the EMT-altered expression of genes encoding the Cer-modifying enzymatic machinery. The data point out that it is important to appreciate that these alterations should be viewed and considered as the EMT-driven reorganization of the entire sphingolipid-related biological network and not be limited to the interpretation as one gene — one outcome. We uncover close associations between the EMT markers and genes comprising the sphingolipid network as well as with pluripotency genes and inflammation-attributed molecules. We show that the EMT-driven mesenchymal/fibroblast-like cell stage is characterized by increased levels of intracellular S1P and LPA accompanied by decreased levels of ceramide species when compared with those lipid levels at the epithelial cell stage. These data strongly support the pro-migratory, anti-apoptotic phenotype of A549 cells that have undergone EMT, and, in conjunction with published data, may suggest their reduced chemoresistance. Considering that the aggressiveness of lung cancer cells is in part defined by their abilities to survive and metastasize, these cell-based conclusions can be related to disease development and patient prognosis.

Yet, given the accessibility of public microarray data sets across various cell conditions, tissues and neoplasms and the power of the GENEVESTIGATOR platform for mapping the entire signature, we herein defined the highest similarity between the sphingolipid/EMT signature and the transcriptional profiles of A549 cell-based models as well as other human epithelial NSCLC cell lines undergoing EMT, and, of particular importance, to the best of our knowledge, for the first time showed that the activation of the sphingolipid-associated EMT program occurs in lung adenocarcinoma tissues of patients with NSCLC. The findings furthermore highlight potential novel regulatory associations between influenza virus and the EMT program in lung cancer with an additional link to the sphingolipid machinery. Moreover, the data suggest that although the herein defined sphingolipid/EMT-attributed mechanism(s) were found to be primarily associated with lung adenocarcinoma, they might further contribute to pathomechanisms of bladder, colorectal, and prostate cancers thus not excluding a common role in pathobiology and/or metastatic process shared between distinct cancer types.

Thus, the highlighted results substantially advance our understanding of the EMT-attributed sphingolipid-associated mechanisms and nominate the sphingolipid machinery among the multifactorial drivers of pathological EMT process and may yield new biomarkers/ potential targets and drug-targeting strategies.

## SUPPLEMENTARY FIGURES AND TABLES















## Abbreviations

S1P: sphingosine 1-phosphate
Cer: ceramide
C1P: ceramide 1-phosphate
HexCer: hexosyl ceramide
SM: sphingomyelin
Sph: sphingosine
S1PR: S1P receptor
LPA: lysophosphatidic acid
LPAR: LPA receptor
EMT: epithelial to mesenchymal transition
TNF: tumor necrosis factor
TGF: transforming growth factor
NSCLC: non-small cell lung cancer
IPA: Ingenuity pathway analysis
MS: mass spectrometry
