# Supplementary material for: Exploring the role of sphingolipid machinery during the epithelial to mesenchymal transition program using an integrative approach
Source: Oncotarget. 2016 Mar 7;7(16):22295–323. doi: 10.18632/oncotarget.7947 (PMC5008362; doi:10.18632/oncotarget.7947)
Supplement: Supplementary file 1 [file oncotarget-07-22295-s001.pdf]

SUPPLEMENTARY FIGURE AND TABLES

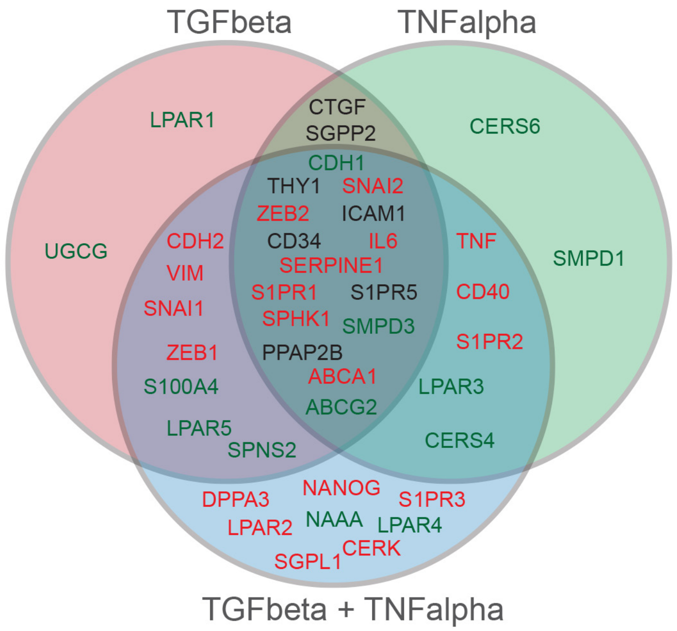

Supplementary Figure S1: Venn diagram displaying common and unique genes across treatment conditions. The sphingolipid/EMT-associated genes showing regulated expression (fold change ≥2 for upregulated and ≤0.5 for downregulated genes at 48 h, Figures 3 and 4) for the indicated treatment conditions — TGFbeta (n=26), TNFalpha (n=24) and TGFbeta + TNFalpha (n=35) — are displayed in three-set Venn diagram. Color code: *red*, upregulated genes; *green*, downregulated genes; *black*, genes showing stimulus-dependent up- or downregulation.

Supplementary Table S1: Correlation analysis of expression data sets derived from three independent experiments. Correlation analysis was performed for each individual gene contributing to the sphingolipid/EMT-associated 35-gene signature, across all time points and treatment conditions. Spearman's correlation coefficients and the corresponding p-values are shown.

See Supplementary File 1

Supplementary Table S2: Correlation matrix. Correlation matrix for expression data sets includes Pearson's correlation coefficients, p-values and FDR-adjusted p-values. Statistically significant FDR-adjusted p-values are highlighted in bold. Color code for statistically significant co-regularities: *red*, correlation coefficient >0.7; *blue*, correlation coefficient ≤0.7.

See Supplementary File 2

Supplementary Table S3: The sphingolipid/EMT signature-linked Canonical Pathways identified by Ingenuity. The ranking is based on the corresponding IPA-based p-value; all significant results are shown (p<0.05); the sphingolipid/EMT signature-derived molecules associated with the corresponding Canonical Pathway are listed.

See Supplementary File 3

Supplementary Table S4: The sphingolipid/EMT signature-linked Upstream Regulators identified by Ingenuity. The ranking is based on the corresponding IPA-based p-value; all significant results are shown ($p<0.05$); the sphingolipid/EMT signature-derived molecules associated with the corresponding Upstream Regulator are listed.

See Supplementary File 4

Supplementary Table S5: Real-time PCR primers. Gene symbol, Gene ID and sequences of forward and reverse primers are indicated.

See Supplementary File 5

Supplementary Table S6: Genes composing the sphingolipid- and EMT-associated multigene signatures. Gene symbol, synonyms, gene name, NCBI accession number, and short functional description are provided.

See Supplementary File 6
